# Immune cell communication networks and memory CD8^+^ T cell signatures sustaining chronic inflammation in COVID-19 and Long COVID

**DOI:** 10.3389/fimmu.2025.1689507

**Published:** 2025-10-22

**Authors:** Hengrui Liu, Zewen Xu, Ilayda Karsidag, Panpan Wang, Jieling Weng

**Affiliations:** 1 Department of Pathology, The Second Affiliated Hospital of Guangzhou Medical University, Guangzhou, China; 2 Guangdong Provincial Key Laboratory of Traditional Chinese Medicine Informatization, Guangzhou, China; 3 San Diego School of Biological Sciences, University of California, San Diego, CA, United States; 4 Department of Traditional Chinese Medicine, The First Affiliated Hospital of Jinan University, Guangzhou, China

**Keywords:** single-cell RNA sequencing, immune cell communication, chronicinflammation, COVID-19, long covid, machine learning, SHAP model

## Abstract

**Background:**

COVID-19, including its post-acute sequelae (Long COVID), is increasingly recognized as involving persistent immune dysregulation and chronic inflammation. Severe and prolonged disease states are often accompanied by sustained cytokine release, immune cell exhaustion, and ongoing cell-cell communication that shapes the inflammatory milieu. Among immune subsets, CD8^+^ T cells play a central role in antiviral defense, yet the molecular mechanisms linking their dysfunction to prolonged inflammation remain incompletely understood.

**Methods:**

We analyzed 73,110 peripheral blood mononuclear cells (PBMCs) from individuals across four disease states (Healthy, Exposed, Infected, and Hospitalized) using single-cell RNA sequencing. Immune cell subsets were annotated, and T cell heterogeneity was profiled. Cytokine and inflammatory scores were calculated to assess immune activation. Differentially expressed genes (DEGs) underwent Gene Ontology (GO) and Kyoto Encyclopedia of Genes and Genomes (KEGG) enrichment analysis. Cell-cell communication was evaluated to map ligand-receptor networks. Additionally, nine machine learning models were trained on a bulk RNA-seq cohort, and the SHapley Additive exPlanations (SHAP) framework was applied to interpret key predictive genes.

**Results:**

Progressive disease severity was associated with a decline in T cell proportions, enrichment of pro-inflammatory myeloid cells, and elevated cytokine expression, particularly IL-32. Memory CD8^+^ T cells showed increased exhaustion and inflammatory scores while maintaining a central position in MHC-I-mediated communication networks. Persistent activation of immune and metabolic pathways, including antigen presentation and oxidative phosphorylation, was observed in prolonged disease states. Seven genes (RPS26, RPS29, RPL36, RPL39, RPS28, RPS21, and CD3E) were identified as strong predictors of chronic immune dysregulation, with the XGBoost model achieving the highest AUC. SHAP analysis confirmed their contributions to disease classification.

**Conclusion:**

This study maps the immune landscape of COVID-19 and Long COVID at single-cell resolution, revealing that persistent immune cell communication, particularly involving memory CD8^+^ T cells, may sustain chronic inflammation beyond the acute phase. The identified molecular signatures offer potential biomarkers and therapeutic targets for mitigating post-viral inflammatory syndromes.

## Introduction

Coronavirus Disease 2019 (COVID-19), caused by Severe Acute Respiratory Syndrome Coronavirus 2 (SARS-CoV-2), has resulted in over 767 million confirmed cases and more than 6.9 million deaths worldwide as of June 2023 (WHO) (1). Although widespread vaccination has significantly reduced infection rates and mortality, the emergence of new variants with enhanced transmissibility and pathogenicity continues to pose serious challenges, making the pandemic far from fully contained (2–4). The impact of COVID-19 is profound, affecting multiple systems and contributing to a substantial disease burden (5). Its clinical manifestations are highly variable, ranging from asymptomatic or mild infections to severe cases and even death, depending on individual immune responses and other risk factors. Studies have identified age, sex, and comorbidities (e.g., hypertension and diabetes) as key determinants of disease progression (6–8). Common symptoms in asymptomatic or mild cases include fever, dry cough, and fatigue, with relatively short disease durations (9). However, in some patients, the condition may rapidly worsen, leading to severe pneumonia, acute respiratory distress syndrome (ARDS), or multi-organ failure (10, 11).

Based on the severity of the disease, COVID-19 can be classified into four states: Healthy (uninfected individuals), Exposed (contacts not yet diagnosed), Infected (confirmed cases without severe progression), and Hospitalized (severe cases) (12). Exposed individuals often exhibit mild or no symptoms but may carry the virus and contribute to its transmission (13, 14). Infected patients, however, experience more significant immune dysregulation, including elevated cytokine levels and mild lymphopenia (15). Hospitalized patients frequently present with a “cytokine storm”, characterized by marked increases in inflammatory cytokines (e.g., IL-6, IL-8, TNF-α), neutrophilia, lymphopenia, and severe immune dysfunction. This excessive and sustained inflammatory response is increasingly recognized as a key contributor to chronic immune dysregulation, driving disease progression and multi-organ damage in Hospitalized patients (16).

Single-cell RNA sequencing (scRNA-seq), a high-resolution gene expression analysis technology, provides a powerful tool for uncovering the immunological mechanisms of COVID-19 (17, 18). The heterogeneity and dynamic changes in immune cells induced by viral infection are difficult to capture using traditional bulk analysis methods. By analyzing gene expression at the single-cell level, scRNA-seq allows for a comprehensive exploration of the functional states and molecular characteristics of specific cell subsets. COVID-19 severity is closely linked to immune dysregulation, particularly inflammation and CD8+ T cell dysfunction. Studies have shown that during the infection phase, the peripheral blood shows significant reductions in T and B cells, coupled with increases in highly inflammatory monocytes and neutrophils (15, 19, 20). Through scRNA-seq, researchers can analyze the transcriptional dynamics of these immune cells in detail, elucidating the mechanisms of cytokine storm and immune paralysis (21). Furthermore, scRNA-seq enables the identification of specific CD8+ T cell subsets, assessing their antiviral capacity and exhaustion status, and offering potential targets for immune restoration interventions (22).

Machine learning models are widely used to analyze complex datasets, particularly high-dimensional data in genomics and transcriptomics. By leveraging diverse algorithmic frameworks and mathematical approaches, these models uncover intricate relationships between patient characteristics and clinical outcomes (23). In clinical applications, models based on the SHAP (SHapley Additive exPlanations) framework are commonly used to quantify the contribution of individual features to predictive outcomes (24, 25). This excessive and sustained inflammatory response is increasingly recognized as a key contributor to chronic immune dysregulation, driving disease progression and multi-organ damage in Hospitalized patients. In this study, we applied SHAP-based models to identify and interpret key molecular markers associated with COVID-19-related immune dysfunction, offering novel insights into inflammatory mechanisms and improving the accuracy of predictive modeling (26).

## Materials and methods

### Collection of RNA sequencing data

Single-cell RNA sequencing (scRNA-seq) data (27) of PBMCs from COVID-19 patients with varying disease severities were retrieved from Data of 197 patients admitted to Yale New Haven Hospital with COVID-19 between 18 March and 5 May 2020 which were previously described (28). This dataset includes samples from four groups: Healthy controls, Exposed individuals (close contacts not yet diagnosed), Outpatients (Infected), and Hospitalized patients (Severe), with two samples per group. The validation Bulk RNA-seq transcriptomic cohort was previously described and generated on the GPL24676 Illumina NovaSeq 6000 platform, encompassing 100 COVID-19 samples and 26 non-COVID-19 controls (29).

### Unsupervised clustering and cell annotation of scRNA data

The raw unique molecular identifier (UMI) count matrix was processed using the R package Seurat (version 5.1.0) and converted into a Seurat object (30). Cells were filtered based on the following criteria: fewer than 1,000 detected RNA molecules, fewer than 200 or more than 10,000 detected gene features, mitochondrial gene proportions (percent.mt) exceeding 20%, and hemoglobin gene proportions (percent.hb) exceeding 90%. After filtering, 73,110 cells were retained for downstream analysis. The dataset was normalized using the ScaleData() function, and the top 2,000 highly variable genes were identified with FindVariableFeatures(). Principal component analysis (PCA) was performed using RunPCA(), selecting the top 10 principal components for dimensionality reduction. t-SNE and UMAP analyses were then conducted using RunTSNE() and RunUMAP(), respectively (31). Clustering was performed with FindNeighbours() and FindClusters(), with the resolution set to 0.3. Cluster-specific marker genes were identified using FindAllMarkers(), applying thresholds of |log2FC| > 0.25 and p-value < 0.05 (32). Finally, cluster marker genes were annotated using the scMayoMap package, integrating scMayoMap Database and lung tissue-specific data (tissue = ‘lung’) (33, 34).

### Analysis of tissue preferences of cell types

To evaluate the distribution preferences of cell types across different tissue states (Healthy, Exposed, Infected, and Hospitalized), the calTissueDist() function from the sscVis package (version 0.1.0) was used to calculate the R_o/e ratio, which quantifies differences in cell type distribution among tissues. The statistical significance of these differences was assessed using the chi-squared test (method = “chisq”), and a cell type ratio matrix associated with tissue states was extracted. The results were visualized as a heatmap created with the ComplexHeatmap package (version 2.18.0). Color gradients were used to intuitively represent changes in R_o/e values, and symbolic annotations were added to heatmap cells to indicate the degree of preference: “+++” for R_o/e > 1, “++” for 0.8 < R_o/e ≤ 1, “+” for 0.2 ≤ R_o/e ≤ 0.8, “+/-” for 0 < R_o/e < 0.2, and “-” for R_o/e = 0. This approach provided a clear visualization of the significant differences in cell type distribution across tissue states (35).

### Single-cell gene set scoring and visualization

To assess the activity of the Cytokine and Inflammatory gene sets at the single-cell level, the AddModuleScore() function was applied to calculate module scores for each cell, which were then mapped onto UMAP plots. Using the ggplot2 package (version 3.5.1), pie charts were generated to illustrate the activity proportions of these two gene sets across different cell types. To compare Cytokine Score and Inflammatory Score among different tissue states (Exposed, Infected, Hospitalized, and Healthy), boxplots were created, and statistical significance was evaluated using the Wilcoxon test. Additionally, T cell subpopulations were analyzed for their scores in Cytokine, Exhaustion, Inflammatory, and Regulatory Effector gene sets, with boxplots used to visualize score comparisons among different T cell subtypes (30).

### T cell subset annotation and gene set scoring

T cell subpopulations were extracted using the subset function and reclustered into eight clusters. The sctype package (version 1.0) was used to annotate these clusters based on gene set scoring of specific T cell markers, identifying Effector CD8+ T cells, Memory CD4+ T cells, Memory CD8+ T cells, Naive CD4+ T cells, and Naive CD8+ T cells (36, 37).

### Single-cell enrichment analysis

To explore the functional characteristics of different T cell subpopulations, differential expression analysis and functional enrichment analysis were performed based on single-cell transcriptomic data. Differentially expressed genes (DEGs) for each T cell subset were calculated using the FindAllMarkers function in the Seurat package, with the top 1,000 genes showing significant upregulation selected based on average fold-change values. The selected DEGs were then subjected to Gene Ontology (GO; biological processes and cellular components) and KEGG pathway enrichment analysis using the compareCluster function in the ClusterProfiler package (38). Gene names were converted to ENTREZ IDs using the org.Hs.eg.db database, and statistically significant enriched terms (p-value < 0.05) were identified. Finally, the enrichment results were visualized as dot plots using the enrichplot package.

### Cell-cell communication analysis

This study employed the CellChat R package, a specialized tool for inferring, analyzing, and visualizing cell-cell communication from single-cell RNA sequencing data (39). The analysis focused on T cell subsets, including Effector CD8+ T cells, Memory CD8+ T cells, Memory CD4+ T cells, and Naive CD8+ T cells. Overexpressed genes and ligand-receptor pairs were identified to calculate intercellular communication probabilities and infer signaling pathway networks. Visualizations, such as circular and bubble plots, were used to depict communication frequencies and strengths among subsets. Key pathways, particularly MHC-I, were analyzed in-depth, revealing the central role of Memory CD8+ T cells in immune modulation. These findings provide critical insights into the mechanisms of T cell-mediated immune regulation and their implications in COVID-19 progression (40).

### Single-cell differential analysis

Differential expression analysis was conducted on Memory CD8+ T cells to investigate gene expression differences across various pathological states (Healthy, Exposed, Infected, and Hospitalized). The analysis employed the Seurat package and the MAST method, comparing each of the three experimental groups to the Healthy control group. The process involved extracting the Memory CD8+ T cell subset, identifying differentially expressed genes (DEGs) using the FindAllMarkers function in Seurat, and filtering genes with adjusted p-values (p_val_adj) less than 0.05 and average log fold-change (avg_log2FC) greater than 0. Results were visualized using the ggplot2 package, generating volcano plots, bubble plots, and lollipop plots to depict the distribution, expression proportions, and commonly upregulated genes among groups (41).

### Machine learning screening

Nine commonly used machine learning methods were applied to model COVID-19-related data, including Linear Discriminant Analysis (LDA), Flexible Discriminant Analysis (FDA), Logistic Regression, Naive Bayes, Support Vector Machine (SVM), Random Forest, Gradient Boosting Machine (GBM), Mixture Discriminant Analysis (MDA), and XGBoost (42). Through multiple randomized experiments assessing AUC in both training and testing datasets, XGBoost demonstrated the best stability and performance. Consequently, XGBoost was selected as the final diagnostic model (43). The xgboost package was employed to train the model, using 70% of the data for training and 30% for testing. Additionally, the rmda package was utilized for Decision Curve Analysis (DCA) to evaluate the model’s net benefits at varying risk thresholds, highlighting its clinical application potential.

### SHAP-based diagnostic model interpretation

The shapviz package was employed to analyze SHAP values, providing interpretability for the XGBoost model. Key diagnostic genes, such as RPS26, RPS29, and RPL41, were identified through feature importance bar plots and beeswarm plots, quantifying their contributions to the prediction results. Furthermore, a force plot was generated for the highest-scoring positive sample, visualizing the positive and negative contributions of individual genes to the prediction outcome.

## Results

### Single-cell transcriptomic analysis of COVID-19 patients

**Figure 1:**
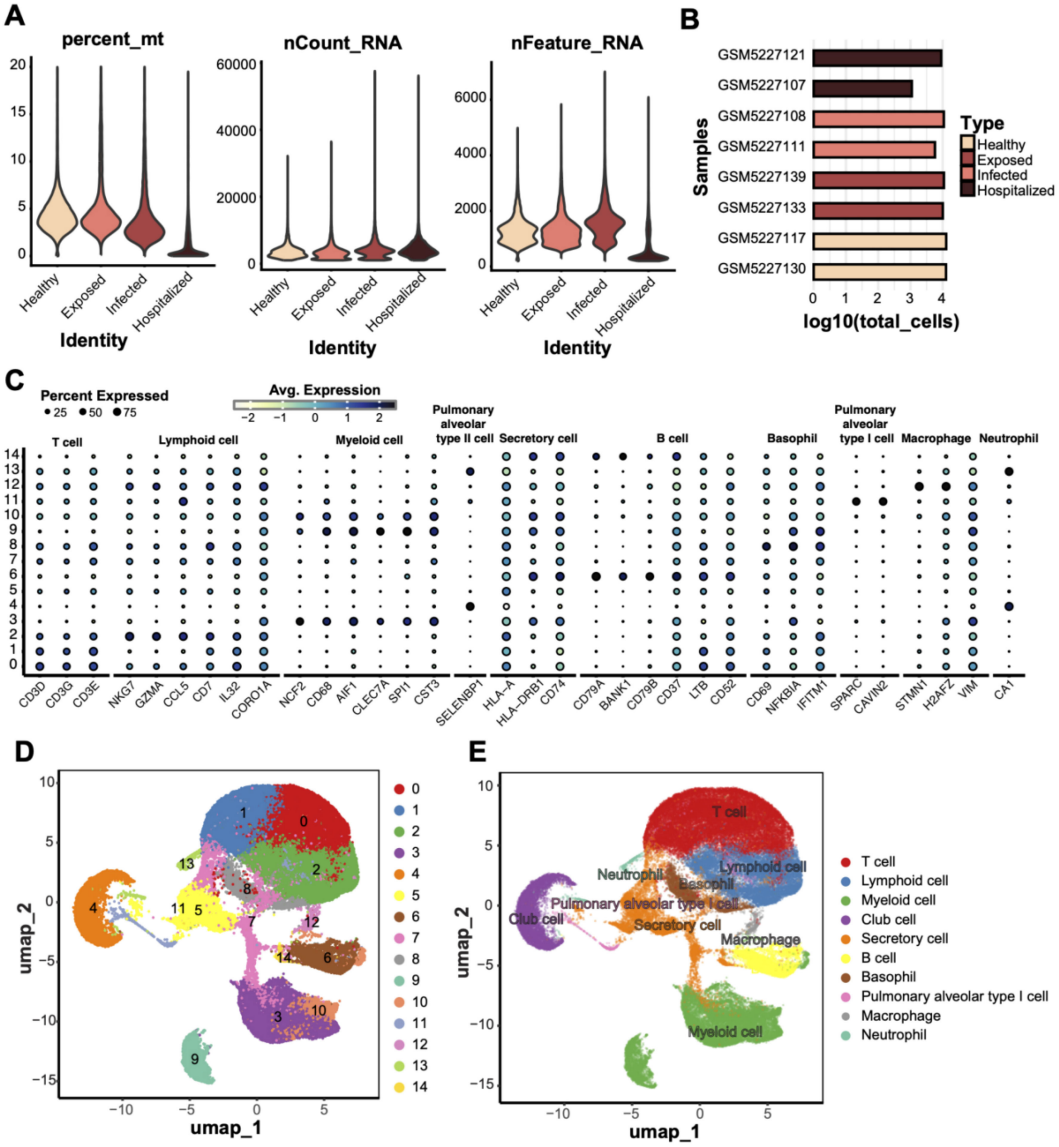
scRNA data quality control and cell type annotation. **(A)** Quality control metrics of scRNA data from four groups (Healthy, Exposed, Infected, Hospitalized), including mitochondrial gene ratio (percent_mt), transcript counts (nCount_RNA), and detected gene numbers (nFeature_RNA). **(B)** Bar plot displaying the total cell counts (log10 scale) per sample, grouped and compared across the four conditions. **(C)** Bubble plot showing the expression of characteristic genes across different cell types; bubble size represents the proportion of cells expressing the gene, and color indicates the average expression level. **(D)** UMAP dimensionality reduction of 14 unsupervised clusters, with each dot representing a cell and colored by cluster ID. **(E)** UMAP clustering annotated with cell type labels, identifying clusters as specific cell types (e.g., T cells, macrophages, and secretory cells).

We obtained peripheral blood mononuclear cells (PBMCs) from eight individuals with confirmed COVID-19, covering a range of disease severities. After quality control procedures, including filtering based on mitochondrial gene content, transcript counts, and the number of detected genes, we retained 73,110 high-quality cells for downstream single-cell RNA sequencing (scRNA-seq) analysis (**Figures 1A, B**). Uniform Manifold Approximation and Projection (UMAP) embedding revealed 15 distinct cell clusters (**Figure 1D**). These clusters were assigned to 10 major immune and epithelial cell types based on canonical marker gene expression (**Figures 1C, E**), including T cells (CD3D, CD3G, CD3E), B cells (MS4A1, CD79A, CD79B), myeloid cells (CD14, LYZ, ITGAM), neutrophils (S100A8, S100A9, CSF3R), macrophages (CD68, CD163, MRC1), secretory cells (SCGB1A1, SCGB3A2), alveolar epithelial type I cells (AGER, HOPX, PDPN), alveolar epithelial type II cells (SFTPC, SFTPB, SFTPA1), plasma cells (MZB1, JCHAIN, TNFRSF17), and basal cells (KRT5, KRT14, TP63).

### Characterization of cell types across disease states

**Figure 2:**
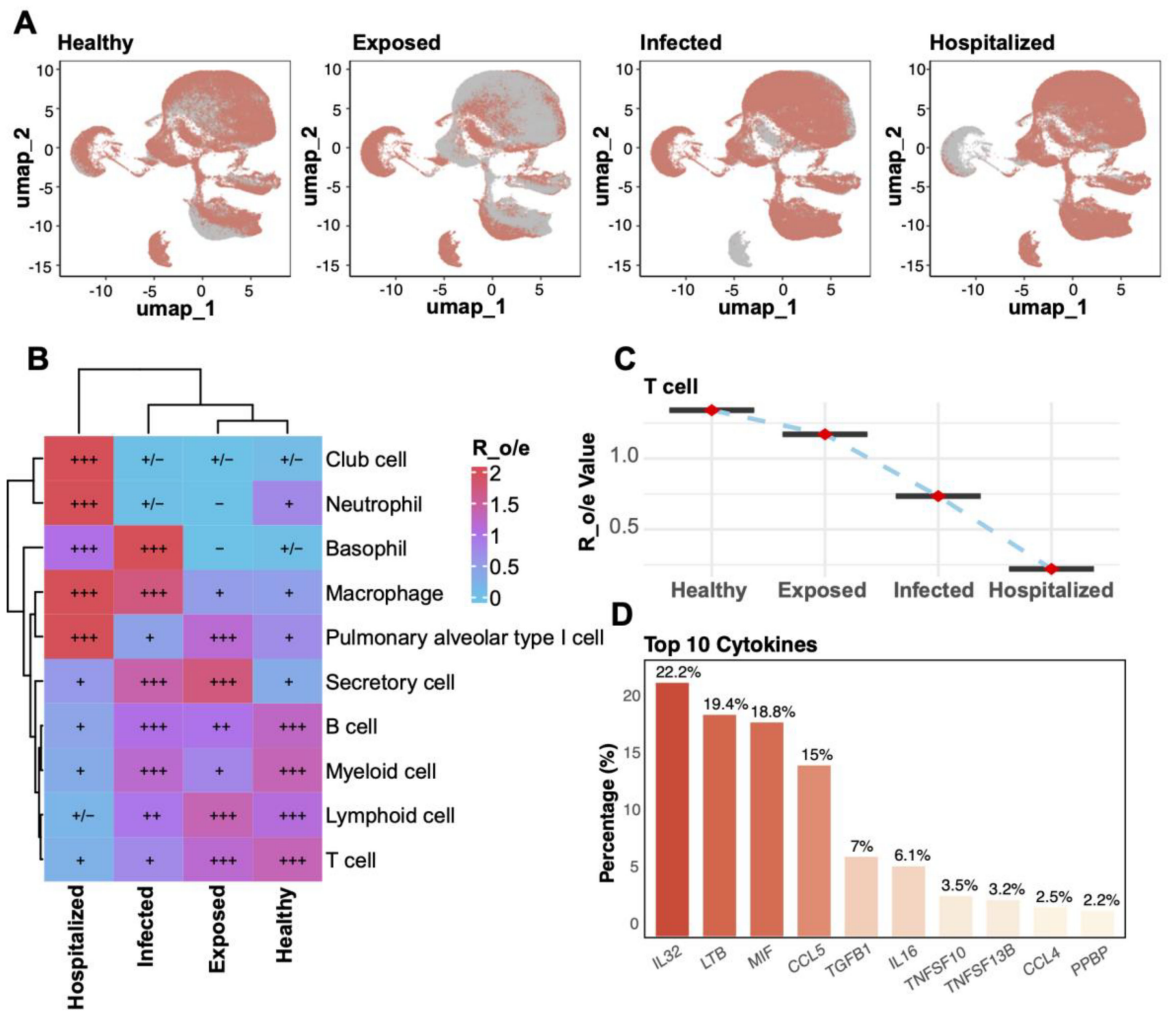
Cell enrichment and key factor analysis across groups. **(A)** UMAP plots for the four groups (Healthy, Exposed, Infected, Hospitalized). **(B)** Heatmap of relative enrichment (Ro/e) for cell types by group. Colors from blue to red reflect enrichment levels; “+” and “–” indicate significant increases or decreases. **(C)** Line plot illustrating the relative enrichment values (R_o/e) of T cells across the four groups, with red points indicating mean values and error bars representing standard deviation. **(D)** Bar plot of the top 10 cytokines’ distribution, highlighting IL32, LTB, and MIF as the most abundantly expressed cytokines across groups.

UMAP analysis compared T cell distributions across four disease states (Healthy, Exposed, Infected, Hospitalized), revealing a progressive decline in T cell proportions with increasing disease severity (**Figure 2A**). Analysis of cell-type enrichment ratios (R_o/e) showed significant enrichment of macrophages, neutrophils, and secretory cells in severe disease states, while T cells were notably depleted in the Infected and Hospitalized groups (**Figure 2B**). T cell enrichment values exhibited a marked decline from Healthy to Hospitalized states (**Figure 2C**), underscoring their potential role in disease progression. Cytokine expression analysis identified IL32, LTB, and MIF among the top 10 cytokines highly expressed in pathological conditions, with IL32 showing the highest expression across all groups, suggesting its pivotal role in inflammatory responses (**Figure 2D**). These findings collectively demonstrate dynamic changes in cell-type composition and highlight their functional implications in disease progression.

### Single-cell transcriptomic analysis of cytokine and inflammatory characteristics across cell types

**Figure 3:**
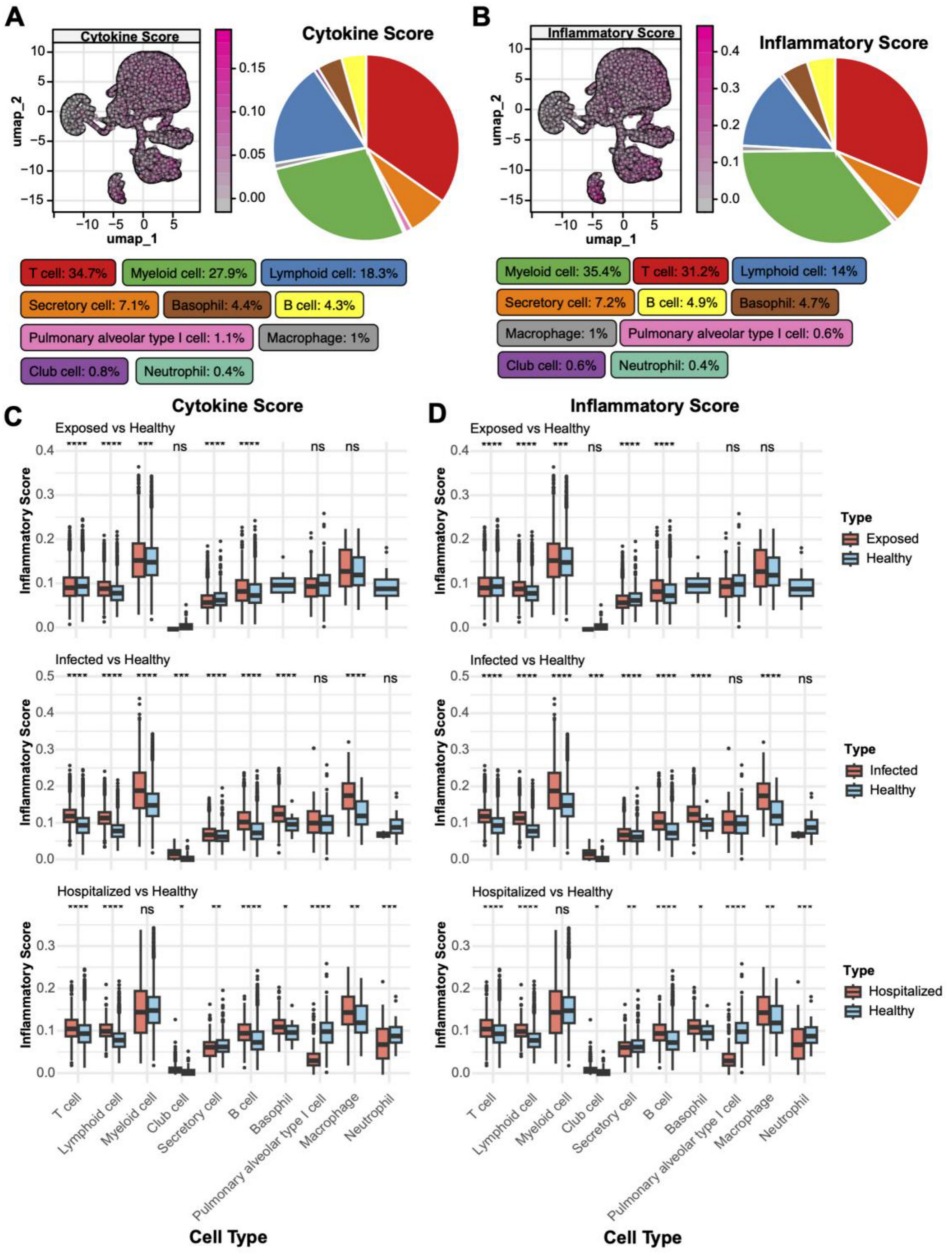
Distribution of cytokine and inflammatory scores. **(A)** UMAP plot of cytokine score spatial distribution, with color gradients indicating score intensity. The pie chart on the right shows the proportion of different cell types within the total population. **(B)** UMAP plot of inflammatory score distribution with a similar pie chart for inflammatory scores. **(C)** Boxplots comparing cytokine scores across cell types in Exposed, Infected, and Hospitalized groups versus Healthy controls (*p < 0.05, **p < 0.01, ***p < 0.001, ****p < 0.0001; ns, not significant). **(D)** Boxplots showing inflammatory score differences among cell types across the groups.

To investigate the role of various cell types in immune regulation and inflammatory responses, we conducted an analysis of cytokine and inflammatory scores. T cells exhibited the highest cytokine scores, constituting 34.7% of the spatial distribution (**Figure 3A**), underscoring their pivotal role in immune responses. Myeloid cells (27.9%) and lymphocytes (18.3%) also displayed significant cytokine activity, indicating their critical involvement in the pathological states. Inflammatory score analysis (**Figure 3B**) revealed that myeloid cells had the highest contribution (35.4%), followed by T cells (31.2%). Comparative analysis across different disease states (**Figures 3C, D**) showed that T cells’ cytokine and inflammatory scores were significantly elevated compared to the Healthy group (p < 0.05), with the highest increase observed in the Hospitalized group. Additionally, neutrophils, macrophages, and secretory cells showed a progressive rise in scores from Exposed to Infected and Hospitalized states, suggesting their amplified activation in exacerbating inflammation. Conversely, alveolar epithelial and basal cells demonstrated minimal variations.

### T cell subtype clustering and functional scoring

**Figure 4:**
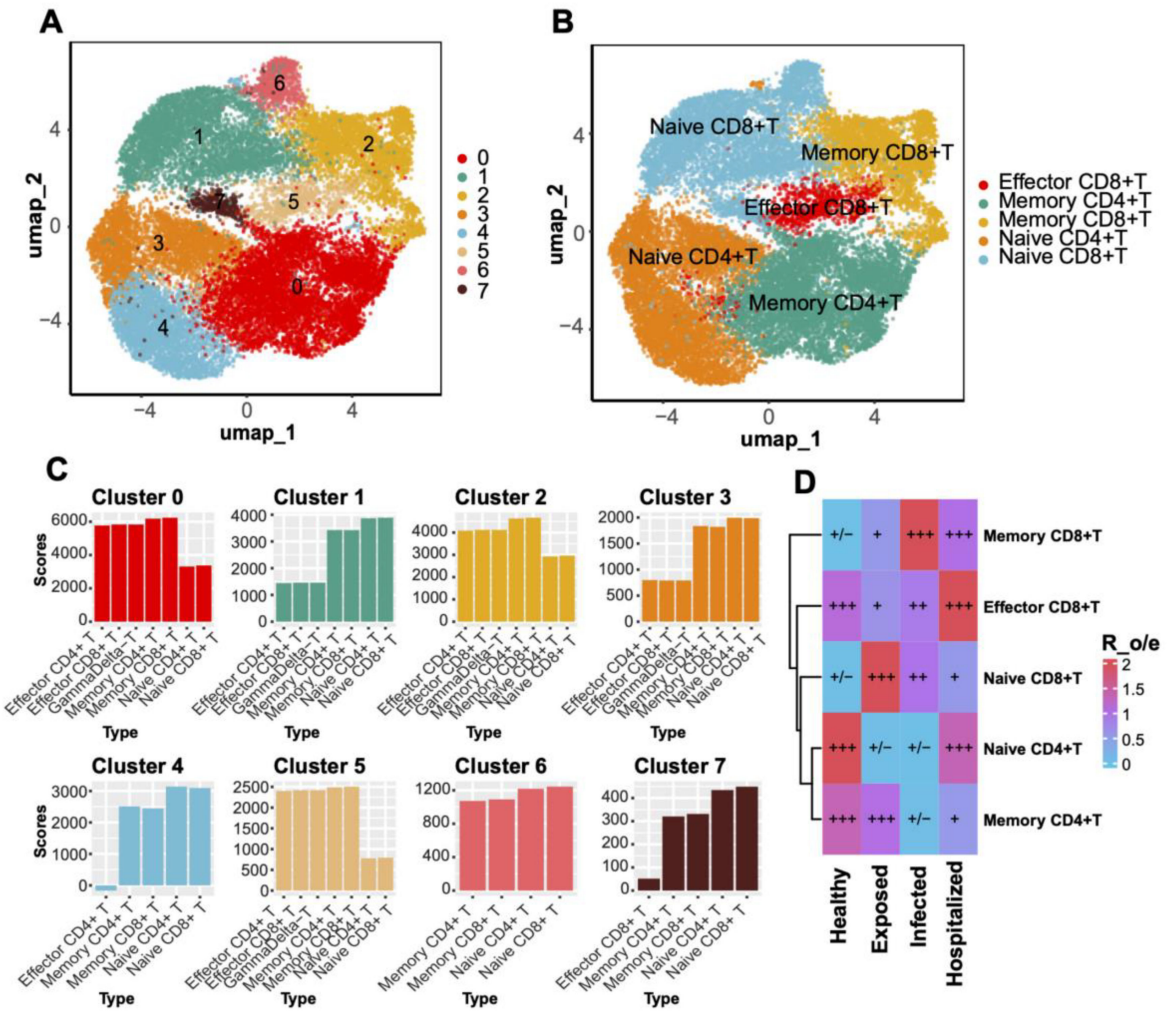
T Cell clustering and subtype distribution. **(A)** UMAP plot displaying T cell clustering into eight distinct clusters. **(B)** Annotated UMAP plot for T cell subtypes. **(C)** Bar plot showing gene set scores for each T cell cluster. **(D)** Heatmap comparing T cell subtype distributions across groups, with Ro/e scores visualized through a blue-to-red gradient. Significant enrichment or depletion is marked with “+” and “–”, respectively.

Through UMAP analysis, T cells were subdivided into eight clusters (**Figure 4A**) and annotated into classical subtypes based on specific markers (**Figure 4C**), including Effector CD8+ T cells, Memory CD8+ T cells, Naive CD8+ T cells, Memory CD4+ T cells, and Naive CD4+ T cells (**Figure 4B**). Enrichment analysis across disease groups (R_o/e values) revealed a higher proportion of naive T cells in Healthy individuals, while effector and memory T cells were significantly enriched in diseased groups, with Memory CD8+ T cells showing the strongest enrichment in the Hospitalized group (**Figure 4D**).

**Figure 5:**
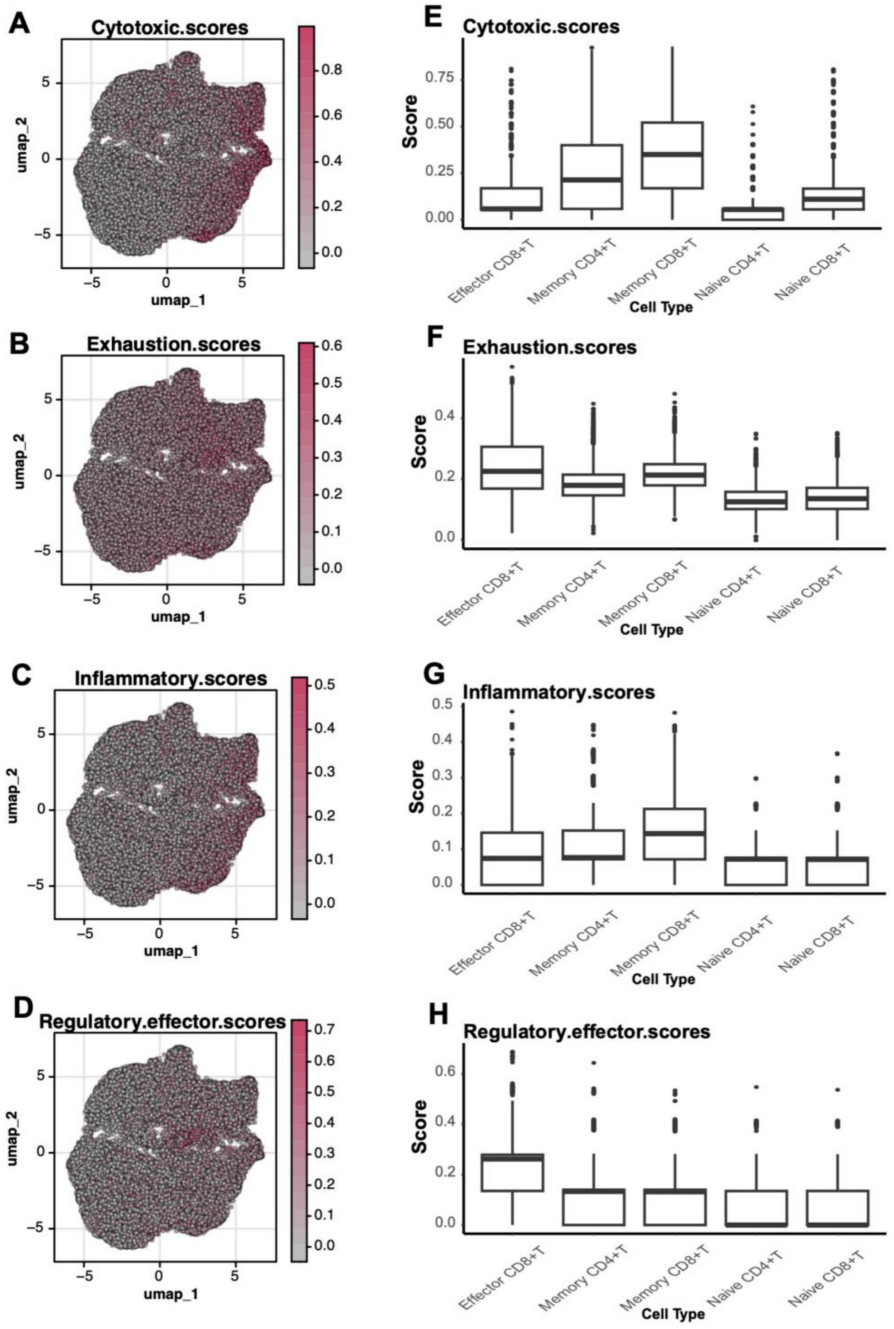
Functional scores of T Cells. **(A-D)** UMAP plots depicting the spatial distribution of cytotoxic (Cytotoxic Scores), exhaustion (Exhaustion Scores), inflammatory (Inflammatory Scores), and regulatory effector (Regulatory Effector Scores) scores in T cells. **(E-H)** Boxplots comparing functional scores across different T cell subtypes.

Functional scoring of gene sets further elucidated subtype-specific characteristics. Effector CD8+ T cells displayed the highest cytotoxic scores, reflecting their critical killing functions during disease progression (**Figures 5A, E**). Memory T cells showed elevated exhaustion and inflammatory scores, suggesting functional impairment during prolonged immune responses (**Figures 5B, F**). Moreover, regulatory effector scores highlighted the role of Memory CD4+ T cells in immune modulation (**Figures 5C, D, G, H**). Collectively, these findings emphasize significant functional heterogeneity among T cell subtypes, particularly the roles of effector and memory T cells in immune responses and pathophysiology.

### Core role of memory CD8+ T cells in cell communication networks

**Figure 6:**
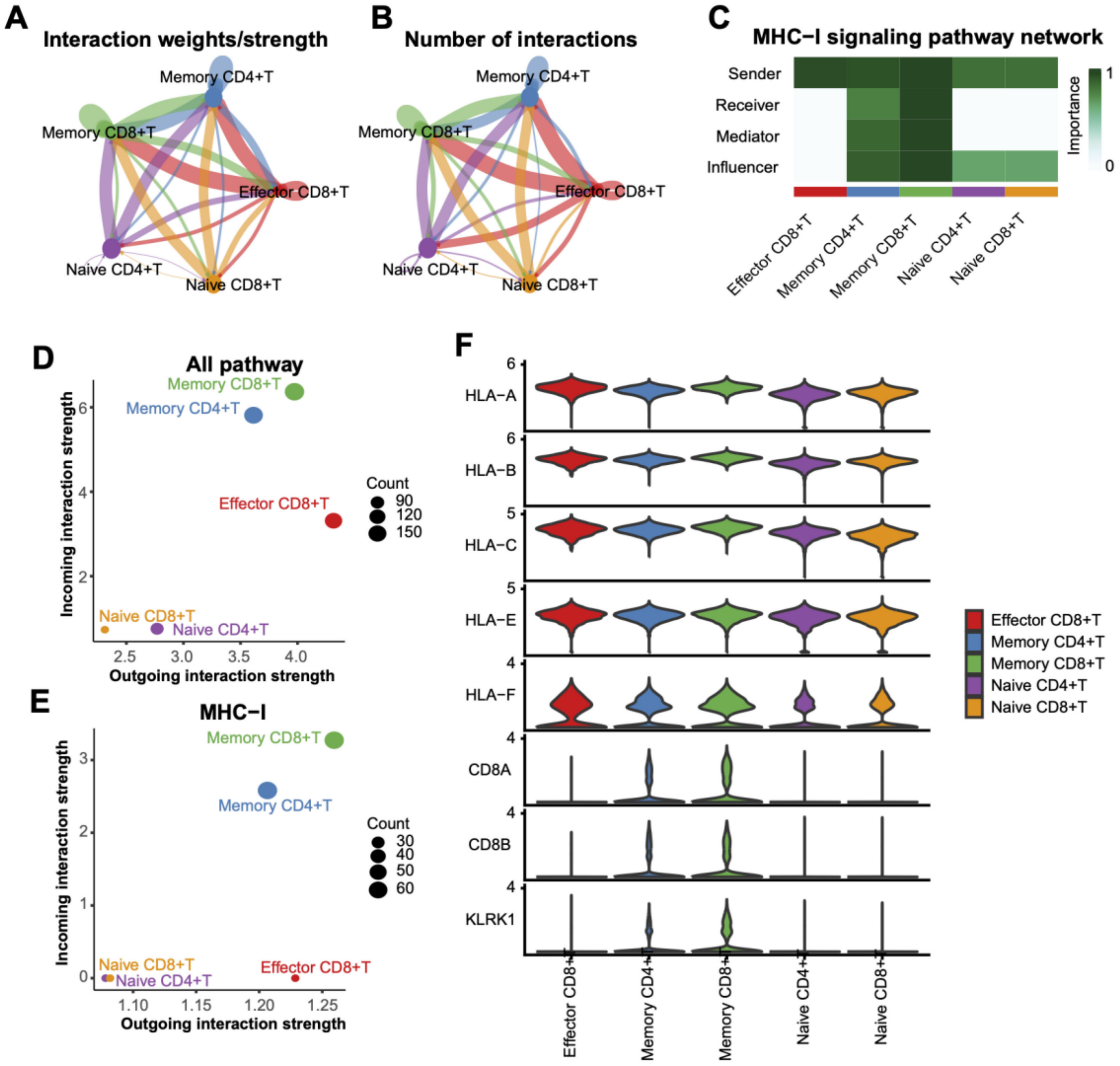
Interaction analysis between T Cell subtypes. **(A)** Interaction weight/intensity network based on all pathways, showing interaction strengths between T cell subtypes. Line color indicates interaction direction, and line thickness represents intensity. **(B)** Network plot of interaction counts between T cell subtypes. **(C)** Role-based heatmap of T cell involvement in MHC-I pathway signaling, with communication importance scaled from 0 to 1. **(D)** Scatterplot of interaction weights across all pathways, with dot sizes representing interaction counts. **(E)** Distribution of interaction weights based on the MHC-I pathway, highlighting the dominance of memory CD8+ T cells in interactions. **(F)** Violin plots of MHC-I pathway-associated gene expression levels across T cell subtypes.

Interaction strength analysis revealed that Memory CD8+ T cells exhibited the highest interaction intensity with other subtypes, such as Effector CD8+ T cells and Memory CD4+ T cells (**Figures 6A, B**). Further exploration of MHC-I signaling pathways underscored the critical involvement of Memory CD8+ T cells (**Figure 6C**). Quantitative analysis of interaction strength (**Figures 6D, E**) highlighted their significant contribution to all pathways, particularly MHC-I signaling. At the transcriptional level, Memory CD8+ T cells demonstrated prominent expression of MHC-I-related genes (e.g., HLA-A, HLA-B, HLA-C) (**Figure 6F**), reinforcing their pivotal role in antigen presentation and immune response regulation.

### Pathway enrichment analysis of memory CD8+ T cells

**Figure 7:**
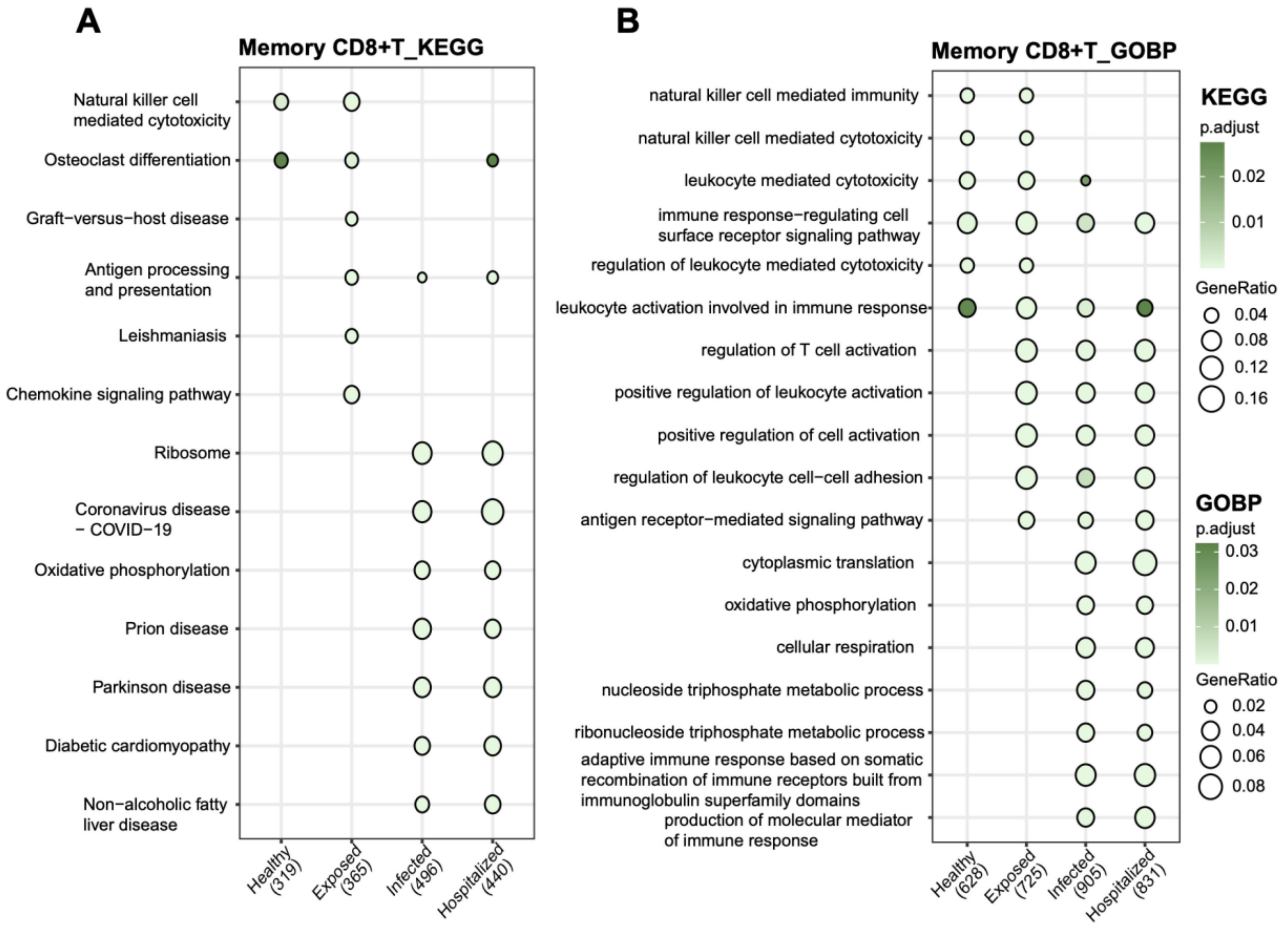
Functional pathway enrichment analysis of memory CD8+ T cells **(A)** KEGG pathway analysis showing functional enrichment in memory CD8+ T cells across groups. **(B)** GOBP analysis of biological process enrichment in memory CD8+ T cells. Bubble size represents the proportion of genes, and bubble color indicates adjusted p-values, with darker green signifying higher significance.

KEGG and GOBP analyses revealed the crucial functional roles of Memory CD8+ T cells under pathological conditions. In KEGG pathways, these cells were significantly enriched in immune-related processes such as natural killer cell-mediated cytotoxicity, antigen processing and presentation, chemokine signaling, and COVID-19-related pathways (**Figure 7A**). Additionally, metabolic pathways like oxidative phosphorylation and ribosome-related pathways were prominently enriched, highlighting their potential role in energy metabolism under active immune states. GOBP analysis further emphasized their involvement in immune regulation, including leukocyte-mediated cytotoxicity, T cell activation, and immune response regulation (**Figure 7B**). These enriched pathways illustrate the essential functions of Memory CD8+ T cells in antigen recognition, immune responses, and inflammation control.

### Differential analysis of single-cell groups

**Figure 8:**
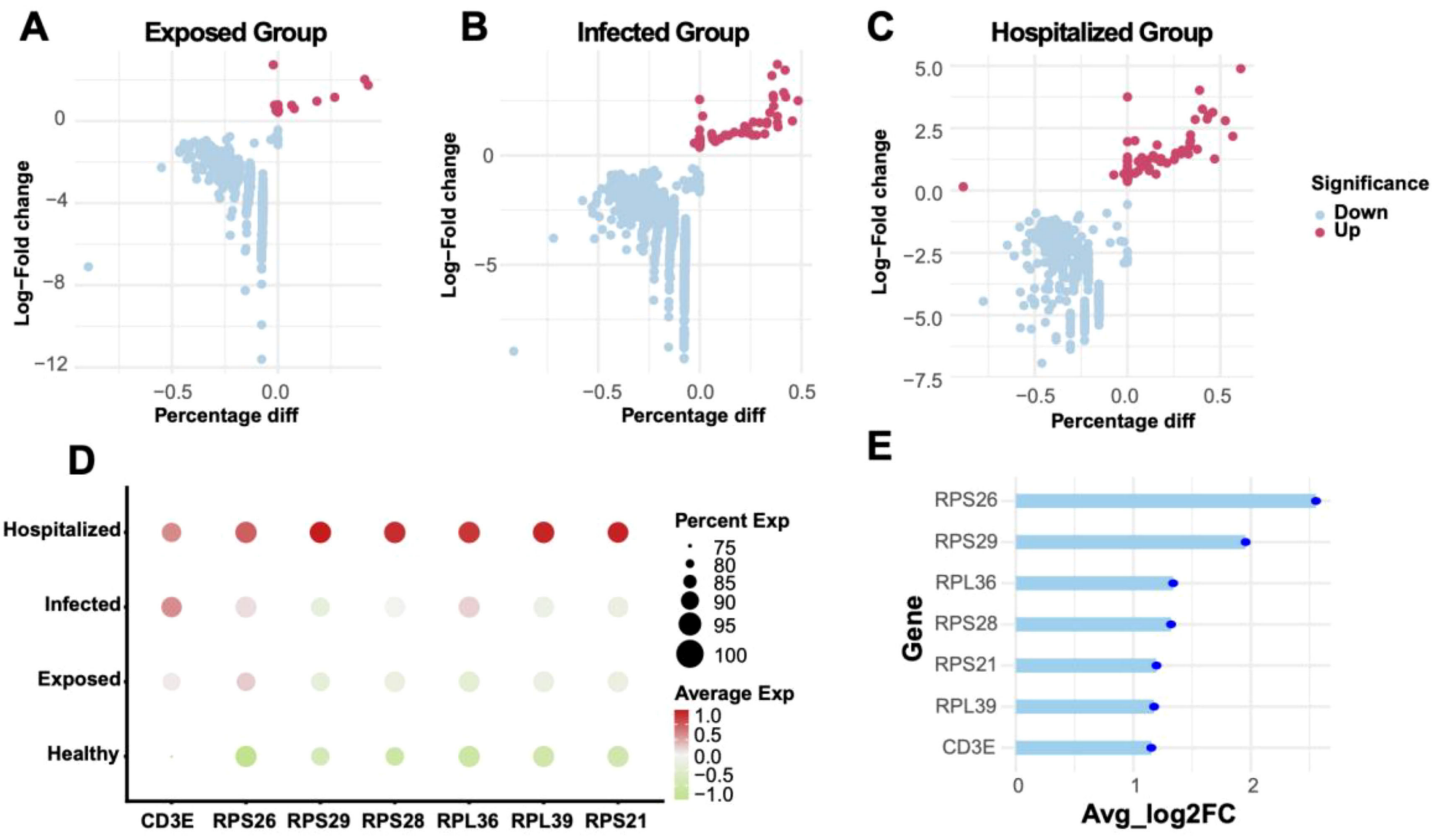
Differential gene analysis and expression characteristics **(A-C)** Volcano plots showing differentially expressed genes (DEGs) between Exposed, Infected, and Hospitalized groups versus Healthy controls. Red dots represent upregulated genes, while blue dots represent downregulated genes. **(D)** Bubble plot of key marker gene expression across groups, with bubble size indicating expression proportion and color denoting average expression levels. **(E)** Lollipop plot displaying the average log2 fold change of significant genes identified across Exposed, Infected, and Hospitalized groups.

By comparing Exposed, Infected, and Hospitalized groups against the Healthy group, volcano plots depicted significant differential gene expression (**Figures 8A–C**). The Hospitalized group exhibited the highest number of upregulated genes, indicating that severe pathological states are associated with substantial transcriptional changes. A cross-group comparison identified seven key genes (RPS26, RPS29, RPL36, RPL39, CD3E, RPS28, RPS21) (**Figure 8E**). Their expression patterns across groups (**Figure 8D**) revealed substantial upregulation in the Hospitalized group, suggesting their involvement in immune responses and protein synthesis during disease progression.

### Multi-model diagnostics and SHAP-based interpretation

**Figure 9:**
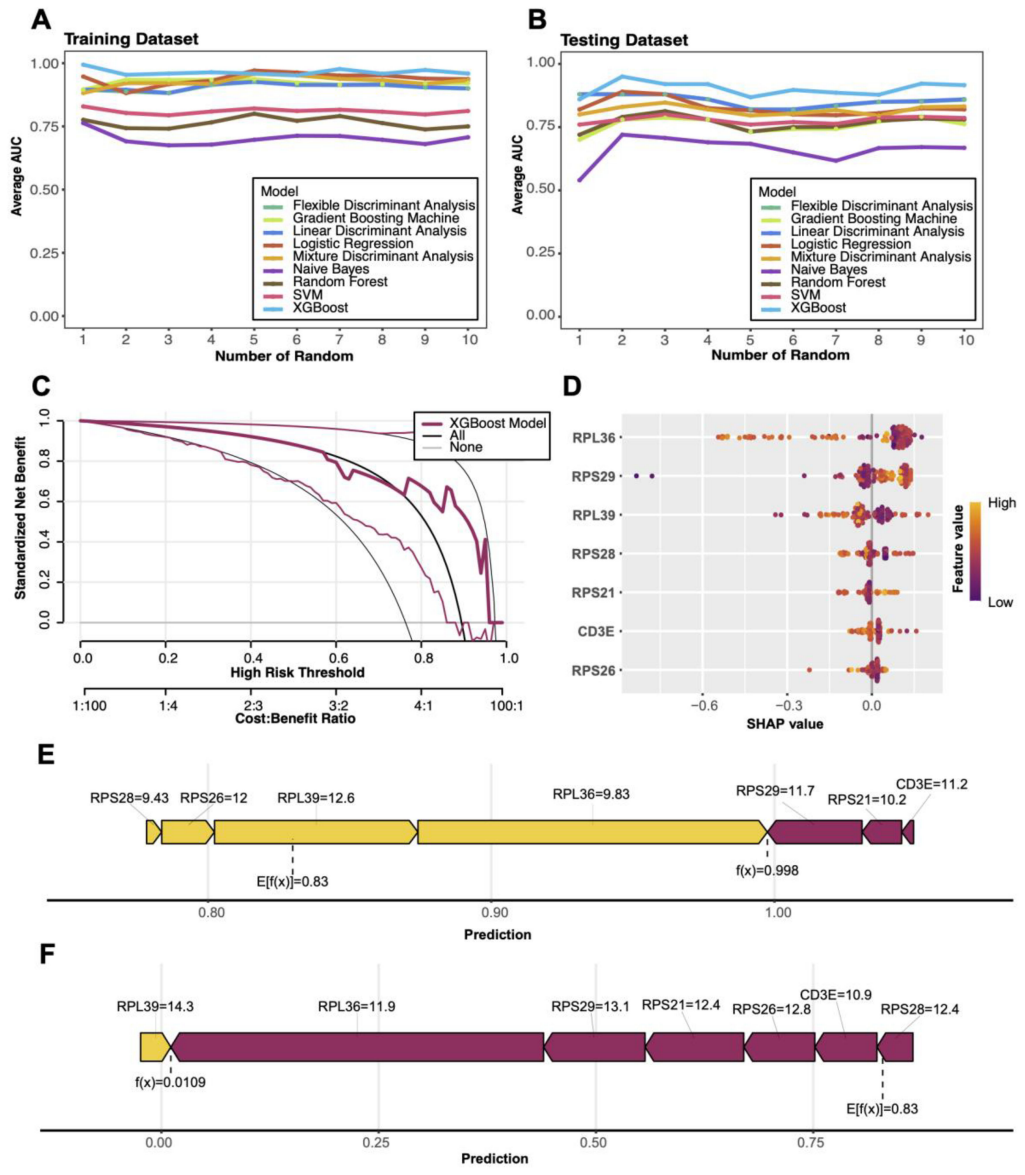
Machine learning model performance and SHAP visualizations **(A, B)** Comparison of average AUC (area under the curve) for nine machine learning models in training and test sets. **(C)** Decision curve analysis (DCA) evaluating the clinical net benefit of the XGBoost model across different risk thresholds. **(D)** SHAP-based feature importance plot highlighting key genes contributing to model predictions, with color indicating feature values. **(E)** Force plot illustrating the predictive mechanism for a positive sample, showing the cumulative contributions of key genes to the model’s output. **(F)** Force plot illustrating the predictive mechanism for a negative sample, showing how key gene features contribute to driving the model output toward a low prediction probability.

Among nine machine learning models, the XGBoost algorithm demonstrated superior performance in both training and testing datasets, achieving the highest average AUC and stable results (**Figures 9A, B**). Decision Curve Analysis (DCA) validated the model’s clinical utility, with standardized net benefits significantly surpassing baseline models (“All” and “None”) across various risk thresholds (**Figure 9C**). SHAP-based analysis identified key predictive genes (e.g., RPS26, RPS29, RPL36), highlighting their significant contributions to disease diagnostics (**Figure 9D**). Additionally, force plots illustrated how these genes cumulatively influenced the prediction outcomes of specific positive samples (**Figure 9E**).

## Discussion

In this study, we systematically characterized the composition and dynamic changes of peripheral immune cells in COVID−19 patients using single-cell transcriptomic analysis. We profiled 73,110 high-quality PBMCs from eight individuals, constructing immune landscapes across four clinical groups: Healthy, Exposed, Infected, and Hospitalized. UMAP clustering and canonical marker annotation identified ten major immune cell types. Among these, T cells were predominant in Healthy donors but exhibited a significant and progressive decline in Infected and Hospitalized patients, a pattern consistent with prior reports describing T cell lymphopenia and dysfunction in COVID−19 cases (44). Conversely, myeloid cells and neutrophils were significantly enriched with increasing disease severity, indicating a shift from adaptive to innate immune responses. Furthermore, expression levels of pro−inflammatory cytokines such as IL−32, LTB, and MIF were markedly elevated in the severe group, with IL−32 persistently upregulated across all disease stages, reinforcing its known pro−inflammatory role in viral infections and lung pathology (45, 46). Despite elevated cytokine and inflammation scores, T cells likely experience functional exhaustion in severe cases, while continuous myeloid cell accumulation may drive amplified inflammatory cascades characteristic of cytokine storms in critical COVID−19 patients (47, 48).

Detailed functional profiling of T cells revealed subtype-specific activity changes across disease stages. Through clustering and gene-set scoring, T cells were classified into eight subpopulations, including effector CD8^+^, memory CD8^+^, naïve CD8^+^, and memory CD4^+^ T cells. Effector CD8^+^ T cells exhibited the highest cytotoxicity scores, whereas memory T cell subsets displayed elevated exhaustion and inflammatory scores, suggesting impaired activation during persistent antiviral response, mirroring T cell fatigue phenomena reported by Snyder et al. and others in COVID−19 immunotypes studies (49). Notably, memory CD4^+^ T cells demonstrated strong regulatory signatures, suggesting an immunomodulatory role. CellChat-based intercellular communication analysis identified memory CD8^+^ T cells as central hubs in the MHC−I-mediated antigen presentation network, with strong interactions involving memory CD4^+^ and effector CD8^+^ subsets. Pathway enrichment further revealed that memory CD8^+^ cells are engaged in both immune-related (e.g., antigen processing, NK-mediated cytotoxicity) and metabolic pathways (oxidative phosphorylation and ribosomal activity), supporting the concept of immunometabolic coordination in activated T cells during viral infection (20).

In additon, through multi-omic integration and XGBoost-based diagnostic modeling, we identified several key genes, RPS26, RPS29, RPL36, RPL39, RPS28, RPS21, and CD3E, with high SHAP values, indicating strong associations with COVID−19 immune pathology. These genes predominantly encode ribosomal proteins involved in protein synthesis and cellular metabolism. Notably, RPS26 has been shown to regulate specific mRNA translation and maintain T cell development and homeostasis *in vivo*, with knockout leading to peripheral T cell deficits (50, 51). CD3E, a core component of the T cell receptor complex, is vital for T cell activation and signal transduction, and its downregulation may impair immune recognition efficiency. These findings not only offer mechanistic insights but also suggest potential therapeutic targets aimed at enhancing T cell function via metabolic or ribosomal modulation. While our study is limited by sample size and lacks direct functional validation, it provides a robust framework for understanding immune reprogramming in COVID−19 and highlights critical cell subsets and molecular markers. Future investigations should prioritize larger cohorts and functional assays to elucidate causal mechanisms linking these genes to T cell dynamics.

Importantly, our findings have direct implications for understanding chronic inflammation in post-viral syndromes such as Long COVID. The persistence of elevated cytokine expression, enrichment of pro-inflammatory myeloid cells, and sustained MHC-I-mediated interactions involving memory CD8^+^ T cells suggest that the immune system remains in a state of low-grade but continuous activation well beyond the acute infection. This prolonged immune communication network—centered on metabolically active yet functionally exhausted memory CD8^+^ T cells—may drive unresolved tissue inflammation. Notably, comorbidities such as diabetes or hypertension may further exacerbate this immune dysregulation, amplifying chronic inflammatory signaling. Our model aligns with emerging evidence that sustained antigen presentation and maladaptive cross-talk between adaptive and innate immune compartments underlie long-term symptoms. Therapeutically, targeting key communication nodes—through modulation of ligand-receptor pathways or restoration of T cell metabolic balance—may help disrupt this cycle and offer new strategies for managing Long COVID and related inflammatory comorbidities.

## Conclusions

This study applied single-cell transcriptomics to map the immune landscape across COVID-19 disease stages, revealing that memory CD8^+^ T cells act as central hubs in sustained immune cell communication networks. These cells, despite signs of functional exhaustion, maintained strong MHC-I-mediated interactions that may perpetuate chronic inflammation, particularly in prolonged disease and Long COVID. Multi-model diagnostic analysis identified seven key genes linked to persistent immune dysregulation, offering potential biomarkers and therapeutic targets. Together, these findings provide mechanistic insight into the maintenance of post-viral inflammatory states and lay the groundwork for precision strategies aimed at restoring immune balance.

## Data Availability

Publicly available datasets were analyzed in this study. This data can be found here: https://www.ncbi.nlm.nih.gov/geo/query/acc.cgi?acc=GSE171555, https://www.ncbi.nlm.nih.gov/geo/query/acc.cgi?acc=GSE157103.
