# Correction: Immune cell communication networks and memory CD8+ T cell signatures sustaining chronic inflammation in COVID-19 and Long COVID

**DOI:** 10.3389/fimmu.2025.1739646

**Published:** 2025-11-13

**Authors:** Hengrui Liu, Zewen Xu, Ilayda Karsidag, Panpan Wang, Jieling Weng

**Affiliations:** 1 Department of Pathology, The Second Affiliated Hospital of Guangzhou Medical University, Guangzhou, China; 2 Guangdong Provincial Key Laboratory of Traditional Chinese Medicine Informatization, Guangzhou, China; 3 San Diego School of Biological Sciences, University of California, San Diego, CA, United States; 4 Department of Traditional Chinese Medicine, The First Affiliated Hospital of Jinan University, Guangzhou, China

**Keywords:** single-cell RNA sequencing, immune cell communication, chronicinflammation, COVID-19, long covid, machine learning, SHAP model

Affiliation 2 “San Diego School of Biological Sciences, University of California, San Diego, CA, United States” and Affiliation 3 “Guangdong Provincial Key Laboratory of Traditional Chinese Medicine Informatization, Guangzhou, China” were erroneously swapped. These affiliations have now been corrected to: 2 “Guangdong Provincial Key Laboratory of Traditional Chinese Medicine Informatization, Guangzhou, China”; and 3 “San Diego School of Biological Sciences, University of California, San Diego, CA, United States”.

The original version of this article has been updated.

